# Optimizing the Strength and Toughness of V/Mo-Modified 0.22C–5.24Mn Steel by Short-Time Partial Austenitization Process

**DOI:** 10.3390/ma17030687

**Published:** 2024-01-31

**Authors:** Haoqing Zheng, Gang Liu, Shuai Tong, Guanqiao Su, Xiaokai Liang, Xinjun Sun

**Affiliations:** 1 State Key Laboratory of Vanadium and Titanium Resources Comprehensive Utilization, Pangang Group, Panzhihua 617000, Chinasuhangjoe@gmail.com (G.S.); 2 Department of Structural Steels, Central Iron and Steel Research Institute Company Limited, Beijing 100081, China; d202110607@xs.ustb.edu.cn (G.L.); landlad@sina.com (S.T.); liangxk@sina.com (X.L.); 3 Institute for Carbon Neutrality, University of Science and Technology Beijing, Beijing 100083, China

**Keywords:** medium manganese steel, short-time partial austenitization, strength and toughness, multiphase hierarchical microstructure, (V, Mo)C precipitation

## Abstract

In order to obtain the good match between yield strength and low-temperature toughness, the short-time partial austenitization (SPA) process was employed for V/Mo-bearing 0.22C–5.24Mn steel. The initial microstructure after intercritical tempering was dual-phase ferrite and reversed austenite (RA), while the final microstructure consisted of ferrite, RA, and secondary martensite (SM) after being subjected to the SPA process. (V, Mo)C with disclike morphology mainly precipitated during intercritical tempering, and the aspect ratio of particles decreased, leading to the appearance of near-spherical morphology. After being subjected to SPA process, the resultant multiphase hierarchical microstructure (three layers: outer layer of ferrite, interlayer of SM, and inner layer of RA) enabled a high yield strength of 1097 MPa, a total elongation of 14%, and an impressive impact energy of 33.3 J at −20 °C. The strengthening contribution of (V, Mo)C precipitation was estimated to be about 108 MPa.

## 1. Introduction

Medium manganese steels (MMS) are promising structural materials, attracting intensive attention due to their superior comprehensive mechanical properties, such as high strength, excellent ductility, and good low-temperature toughness, attributing to the introduction of reversed austenite (RA) [1,2,3,4]. RA is formed by the intercritical annealing or tempering (termed as IT or IA) process, where the specimen is heated to α + γ two-phase region for C and Mn partition [5,6]. The treating temperature and preservation duration will affect the stability of RA [7,8], which can be applied to tailor the mechanical properties by the transformation-induced plasticity (TRIP) effect [9]. To obtain an admirable product of tensile strength and total elongation, there are many new types of treatment processes for tailoring RA to induce the positive TRIP effect. Sadeghpour et al. [10] proposed a new combinatorial processing route, low-temperature prior deformation and high-temperature annealing partitioning, to achieve an ultrafine-grained, multiphase microstructure and stable austenite in an MMS steel. A deformed and partitioned process was treated by Liu et al. [11] to tailor the lamellar structure of austenite and martensite to realize high fracture toughness under the yield strength of 2 GPa. A combination of cold rolling and intercritical recrystallization annealing is often adopted to control the reversed austenite with different stabilities [12]. However, studies on the process to tailor the MMS steel with high yield strength and good low-temperature toughness are rarely reported.

In MMS dual-phase steel, ferrite possesses high toughness and ductility [13], whereas RA contributes to good plasticity and toughness [14]. Furthermore, the RA-induced TRIP effect during deformation can significantly increase the work hardening capability [15], contributing to good synergy of strength and ductility. Unfortunately, both ferrite and RA are soft phases. Although the initial microstructure was fully martensite for low-carbon MMS [16], the supersaturated C atoms diffuse into RA during IT [17], leading to the formation of ferrite [18]. Compared to ferrite, RA is easier to deform at lower strain level, so MMS subjected to the traditional IT or IA process has a low yield strength. For engineering structures, the good match between yield strength and low-temperature toughness shows more importance than the match between tensile strength and total elongation. Yield strength is the ability to suppress deformation, while toughness is the ability to inhibit crack propagation. Therefore, the MMS with low yield strength is limited in wide application.

Many attempts have been utilized to increase the yield strength of MMS; among them, the most crucial methods lie in the introduction of nanoscale particles and short-time partial austenitization (SPA) treatment [19,20]. Due to the affinity difference between alloying elements and C, i.e., Ti, Nb, and V, the carbides would precipitate at different ranges of temperature, which is also affected by the solubility of alloying elements. Importantly, the temperature range of Mn partition is usually the optimal precipitation temperature range. With higher solubility in α-Fe, microalloyed V tends to precipitate with C at lower temperature compared with Ti and Nb. The carbides formed at low temperature have small size and then possess a considerable strengthening effect [21,22]. Unfortunately, the ripening rate of VC is higher compared to TiC and NbC. Molybdenum is an element that not only promotes the precipitation of the MC phase, but also inhibits the ripening rate. Meanwhile, Mo could also increase the additional strengthening effect. Therefore, the coupling addition of a microalloying element and Mo is the development trend of MMS. Alternatively, the SPA treatment is another effective route to strengthen MMS by grain refinement and the introduce of hard secondary martensite (SM). Although many efforts have been made, the mechanism of the SPA process has been seldomly investigated in microalloyed MMS. Therefore, in this work, 0.22C–5.24Mn steel microalloyed by 0.26Mo and 0.15V was designed. The SPA process was applied for the steel, and the phase transformation mechanism and strengthening contributions were analyzed.

## 2. Experimental Materials and Procedure

**Figure 1:**
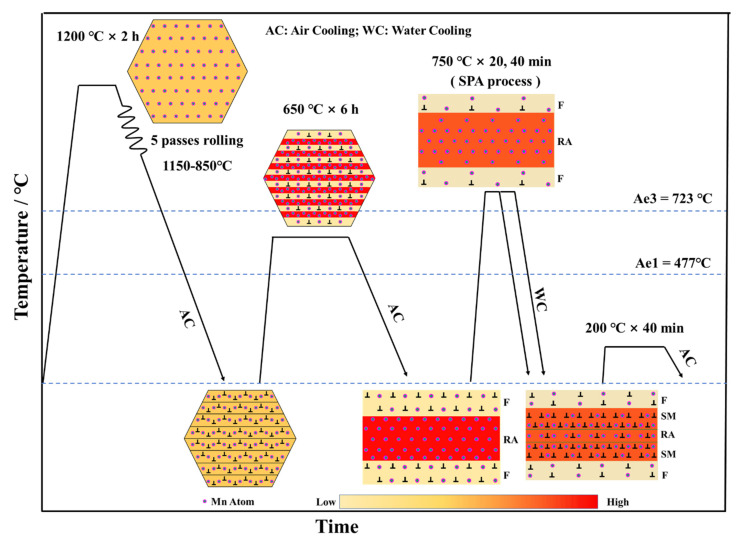
Schematic diagram of hot rolling, multistep heat treatment process, and phase transformation (AC: air cooling; WC: water cooling; F: ferrite; RA: reversed austenite; SM: secondary martensite; ⊥ represents the dislocations).

The nominal composition of the studied steel is Fe–0.22C–0.31Si–5.24Mn–0.26Mo–0.15V–0.026Ti (wt.%). The steel was smelted in a 50 kg vacuum induction furnace. After casting, the steel was forged into a billet with cross-section dimensions of 150 mm and 60 mm. The steel was rolled from 60 mm into a final thickness of 20 mm by several passes, with air cooling to ambient temperature. Subsequently, the hot-rolled steel was subjected to a multistep heat treatment, as shown in Figure 1. The steel was reheated to 650 °C to trigger the RA transformation to dissolve cementite. The heat preservation time was about 6 h for adequate Mn partition. The SPA process was performed at 750 °C with total time of 20 and 40 min in the furnace (namely, SPA20 and SPA 40) after 650 °C IT (referred as IT650), respectively, subsequent with water cooling to room temperature. In order to eliminate quenching stress, a low-temperature process of 200 °C for 40 min was performed.

Standard round tensile specimens with the gauge length of 50 mm, diameter of 10 mm, and total length of 110 mm were prepared from steels along the rolling direction according to our national standard: GB∕T228.1-2021. These specimens were performed at a strain rate of 0.5 mm/min at room temperature using a WE-300 hydraulic tensile testing machine (Jinan Chenxin testing machine Manufacturing Co., Ltd., Jinan, China) for mechanic properties. Charpy V-notch (CVN) specimens with 10 mm × 10 mm × 55 mm were also prepared along the rolling direction and performed on a JBN-300B impact machine (Xi’an Mingkesi Checking&Measuring Equipment Co., Ltd., Xi’an, China) at −20 °C. There were two samples for tensile properties, while the low-temperature impact energies were determined by three samples.

Microstructural characterization was conducted using an FEI Quanta 650 field emission scanning electron microscope (SEM, Hillsboro, OR, USA). Samples for SEM observation were polished and etched in a solution of 4% nital. The OXFORD NordlysNano detector was used for electron backscattered diffraction (EBSD, Oxford Instruments, Abingdon, UK) examination, with the step size of 0.05 μm. Slices with 300 μm thickness were cut and machined to 50 μm thickness, and were subjected to twin-jet electrolytic polishing under a constant current of 30 mA. The prepared samples were observed by FEI TECNAI G^2^ 20 transmission electron microscopy (TEM, Hillsboro, OR, USA) with 200 kV voltage. A Co-Kα target was used for X-ray diffraction (XRD, Bruker, Karlsruhe, Germany) to detect matrix phases after the removal of the samples’ surface residual stress. The scanning step size was 0.02° and the scanning speed was 76.8°/min. The volume fractions of phases were estimated by the following equation [20]:

(1) $$V_{\gamma }=\frac{1.4I_{\gamma }}{I_{\alpha }+1.4I_{\gamma }}$$

where *I_α_* and *I_γ_* are the diffraction peaks of α-Fe and γ-Fe.

## 3. Results

### 3.1. Microstructure Characterization

**Figure 2:**
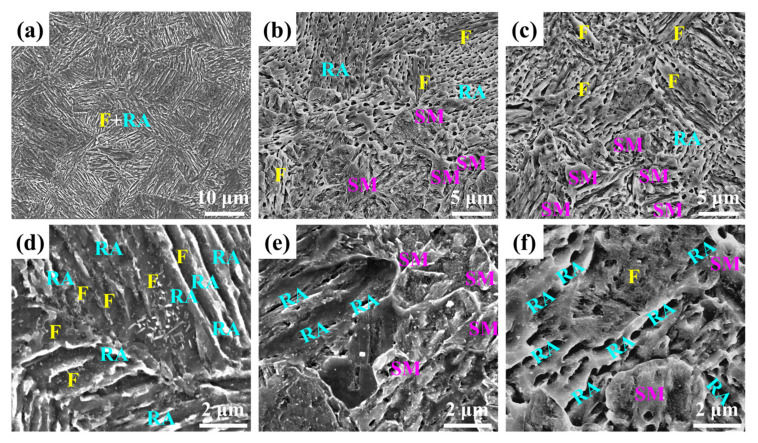
SEM images: (**a**) and (**d**) IT650; (**b**) and (**e**) SPA20; (**c**) and (**f**) SPA40. (F: ferrite; RA: reversed austenite; SM: secondary martensite).

Figure 2 shows the SEM microstructure characterization of samples in different stages. The microstructure of IT650 consisted of RA and tempered martensite. Considering the partition time of 6 h, the carbon atoms diffused from martensite into RA, and no cementite formed, so the tempered martensite is so-called ferrite, intercritical ferrite. After being subjected to the SPA process, the microstructure contained RA, ferrite, and secondary martensite (SM), where the block structure was SM, the lathlike structure was ferrite, and the corrosion hole was RA. The opinion that corrosion holes originate from a weak interface boundary between RA and ferrite or SM comes from our previous reports [6,17]. Moreover, there is lathlike RA distributed along martensite laths, while RA is lathlike and granular after being subjected to the SPA process.

**Figure 3:**
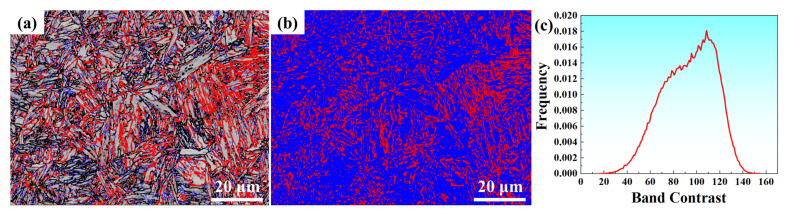
EBSD images of IT650: (**a**) band contrast image of ferrite inserted by RA in red color; (**b**) phase image: blue color is ferrite and red color is RA; (**c**) band contrast curve of ferrite. Coarse black line and fine blue line represent the high-angle grain boundary (>15°) and low-angle grain boundary (2~15°), respectively.

Further microstructure from EBSD shows that the microstructure after being subjected to IT at 650 °C possessed a mixed microstructure of ferrite and RA, as shown in Figure 3. The RA was mainly distributed among martensite lath and martensite substructure boundaries. The microstructure fits well with the result in Figure 2a. The content of RA was about 25.1%, tested by EBSD.

**Figure 4:**
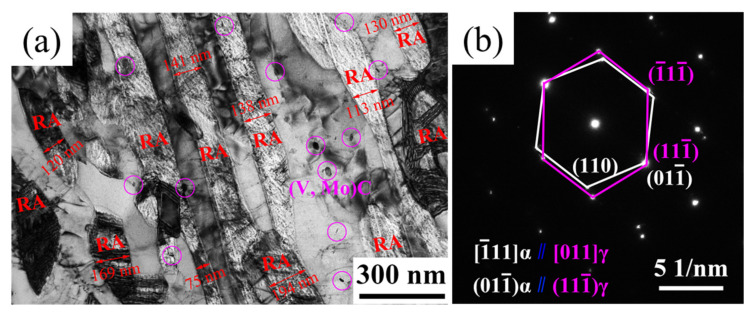
TEM images of IT650: (**a**) bright field image; (**b**) selected area electron diffraction pattern showing Kurdjumov–Sachs orientation relationship.

TEM characterization shows that the mean RA width was about 137 nm, while the mean martensite lath width was about 500 nm (Figure 4a). The mean RA width was about 27.4% of the mean martensite lath. Figure 4b presents the Kurdjumov–Sachs orientation relationship between martensite lath and RA [6,7]. Some stacking faults appeared in the RA lath. Furthermore, plenty of dispersed particles were observed and many of them were disclike. Interestingly, some (V, Mo)C particles were located in the RA/ferrite interface boundary, pinning the migrating velocity of the γ/α interface [23].

**Figure 5:**
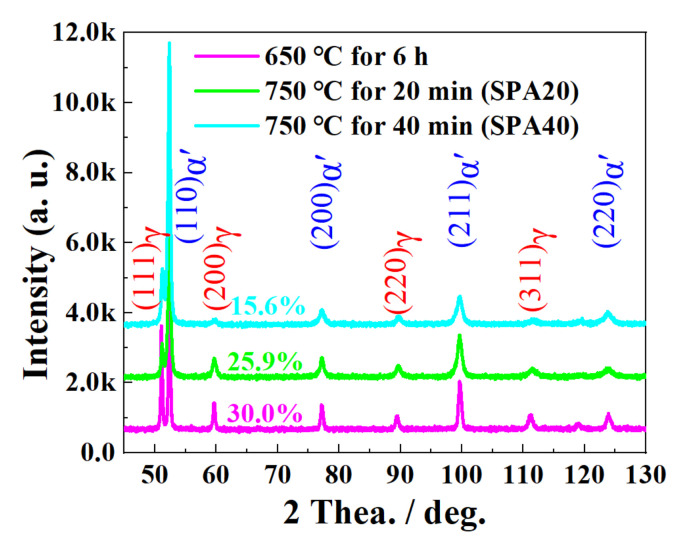
XRD line spectra of samples at different stages.

As shown in Figure 5, the estimated content of RA was about 30.0%, 25.9%, and 15.6% for IT650, SPA20, and SPA40, respectively. The result showed that part of the RA of IT650 transformed in to martensite. Limited by the measurement accuracy of EBSD, the RA content detected by XRD was used for the subsequent strengthening mechanism analysis. Furthermore, the width of diffraction peaks of α-Fe and γ-Fe increased after being subjected to SPA treatment, indicating the higher dislocation density in austenite and martensite [24]. Another phenomenon was located where the diffraction peaks of γ-Fe shifted toward the right, implying the lower carbon content in RA [1].

**Figure 6:**
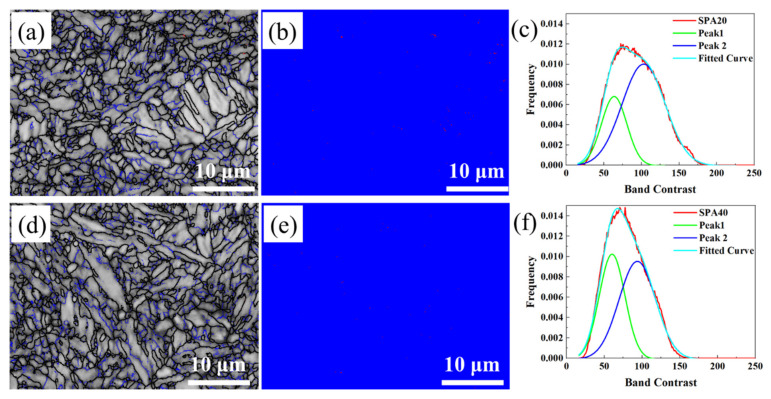
EBSD images: (**a**) band contrast image, (**b**) phase image, and (**c**) band contrast curves of SPA20; (**d**) band contrast image, (**e**) phase image, and (**f**) band contrast curves of SPA40. (**c**,**f**) The band contrast peak of SPA20 and SPA40 was divided into two peaks of F and SM. Coarse black lines and fine blue lines represent the high-angle grain boundary (>15°) and low-angle grain boundary (2~15°), respectively.

The EBSD characterizations of SPA20 and SPA40 are listed in Figure 6. Limited by the poor calibration rate of hard martensite with high-density dislocations, RA was almost not detected. According to the difference between ferrite and fresh martensite, the peak-differentiating method was used to divide the microstructure into ferrite and SM [25,26]. According to the curves of peak1 (SM) and peak2 (ferrite), the estimated content of ferrite was 53.8% and 53.6% for SPA20 and SPA40, while the content of SM was 20.3% and 33.8%, respectively.

**Figure 7:**
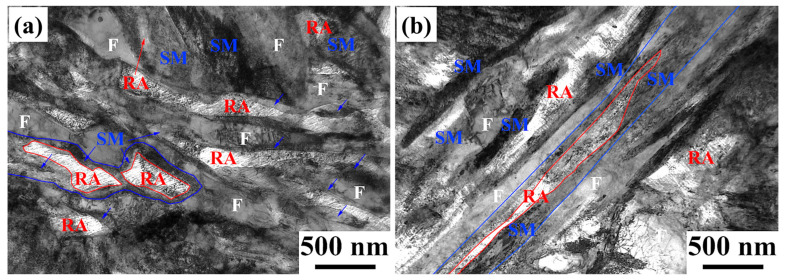
TEM images of (**a**) SPA20 and (**b**) SPA40. (F: ferrite; RA: reversed austenite; SM: secondary martensite).

TEM images show that the microstructure was a mixed complex-phase structure after being subjected to the SPA process (Figure 7). The microstructures of three-layer microstructures were obtained: ferrite (outer layer), SM (interlayer), and RA (inner layer). The contents of them are consistent with the results of EBSD analysis (Table 1).

**Figure 8:**
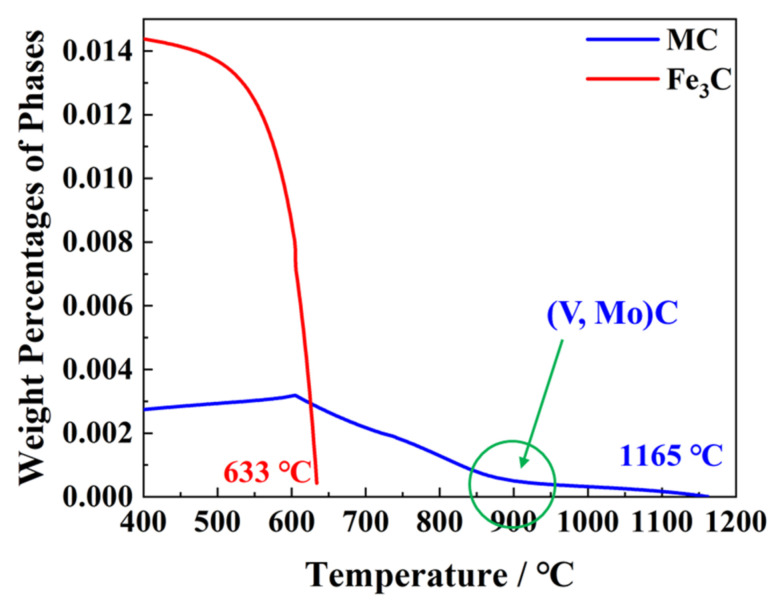
The precipitation contents varying with temperature, calculated by Thermo-Calc.

**Figure 9:**
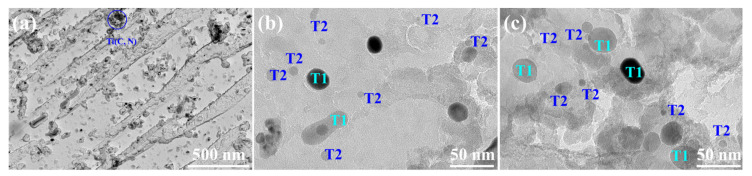
TEM images of carbon extraction replicas of (V, Mo)C particles: (**a**) IT650, (**b**) SPA20, and (**c**) SPA40.

The content of both (V, Mo)C and cementite varying with temperature was calculated by Thermo-Calc with TCFE10.0 database (Figure 8). The completely dissolved temperature of cementite is 633 °C, while it is 1165 °C for (V, Mo)C precipitation. It was the addition of microalloying Ti that increased the completely dissolved temperature of (V, Mo)C precipitation. In actuality, the addition of Ti almost precipitates with N at high temperature so that V carbides mainly precipitate at about 900 °C, where the slope of the precipitation curve changes. Therefore, it could be deduced that the precipitation was mainly (V, Mo)C below 900 °C.

(V, Mo)C particles before and after being subjected to the SPA process are displayed in Figure 9. The results show that the (V, Mo)C precipitates are disclike; some of them are parallel to the martensite and RA length direction, showing the parallel orientation relationship. Once subjected to the SPA process, the particle morphology of (V, Mo)C changed from the disclike shape to a near-spherical shape, which relied on the different orientation relationship between (V, Mo)C and ferrite and austenite. Furthermore, the number density did not vary, indicating that the (V, Mo)C almost precipitated when tempered at 650 °C. The particles, marked by blue in Figure 9a, usually had large sizes due to the fast diffusivity at high temperature, so they provided limited strengthening contribution [27]. There are two types of (V, Mo)C particles after being subjected to the SPA process: Type 1, with bigger size precipitated during rolling and Type 2, with smaller size precipitated during the IT and SPA processes, named T1 and T2, respectively (Figure 9b,c).

### 3.2. Mechanical Property Characterization

**Figure 10:**
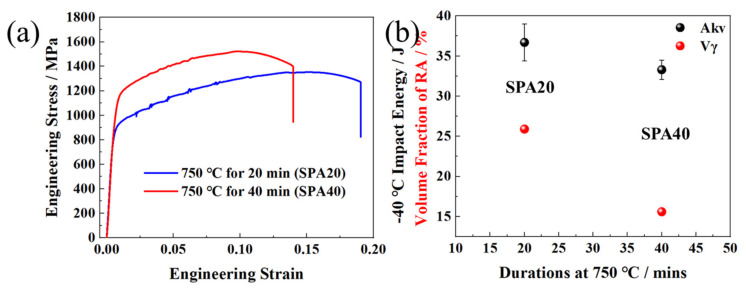
(**a**) Engineering stress and strain curves of SPA20 and SPA40; (**b**) relationship between −20 °C impact energy and volume fraction of RA.

The engineering stress and strain curves of SPA20 and SPA40 are shown in Figure 10a. The yield strength increased from 882 MPa to 1097 MPa, with the total elongation shrinkage from 19.0% to 14.0% of SPA20 and SPA40 (Table 2). The tensile strength was 1341 MPa and 1521 MPa, respectively. The −20 °C impact energies were 36.7 and 33.3 J for SPA20 and SPA40, respectively (Figure 10b and Table 2). The results indicate that the yield strength was optimized by the SPA process with little loss of ductility and toughness.

## 4. Discussion

### 4.1. Phase Transformation Mechanism Analysis

Considering the high hardenability of 0.22C–5.24Mn steel, the initial microstructure was single martensite after rolling. During intercritical tempering at 650 °C for 6 h, the partition of both C and Mn appeared, implying the RA transformation presented. Both Figure 2a and Figure 3 show that the RA was mainly displayed along martensite lath, and the morphology of RA was also lathlike. Although other martensite substructure boundaries could also be regarded as the nucleation sites of RA, the number of martensite lath boundaries is more than other substructure boundaries [6,7]. Therefore, the plane model of nucleation was used to simulate the kinetics of RA transformation. The mean martensite lath width was estimated to be about 500 nm, and the initial RA width was about 4 nm.

**Figure 11:**
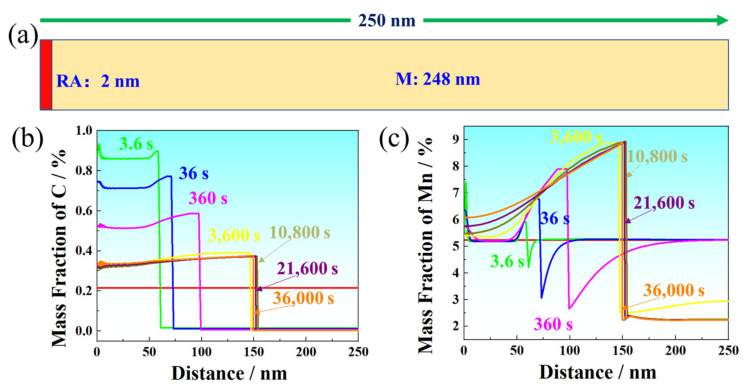
(**a**) Schematic diagram of the diffusion couple applied for DICTRA simulation of IT650. (**b**,**c**) Element concentration profiles of C and Mn near the γ/α interface, showing the moving γ/α interface.

The schematics of the semidiffusion couple applied for DICTRA simulation of IT650 are presented in Figure 11. As the transformation time increased, the α/γ interface migrated from lath boundaries toward the martensite interior. Meanwhile, the carbon concentration gradient across the α/γ interface decreased and some spikes disappeared. Different from the distribution of C, the distribution of Mn was uneven, especially in γ-Fe. Although the phase transformation time increased to 21,600 s, the distribution in γ-Fe was not uniform in γ-Fe. However, the distribution of Mn was uniform as the phase transformation time was above 3600 s. The results show the higher diffusion velocity of C than Mn, and the higher diffusion velocity in α-Fe than γ-Fe. Furthermore, the α/γ interphase migrated in reverse when the phase transformation was above 3600 s. After intercritical tempering at 650 °C for 6 h, the microstructure consisted of ferrite and RA, which was estimated to be about 30%.

**Figure 12:**
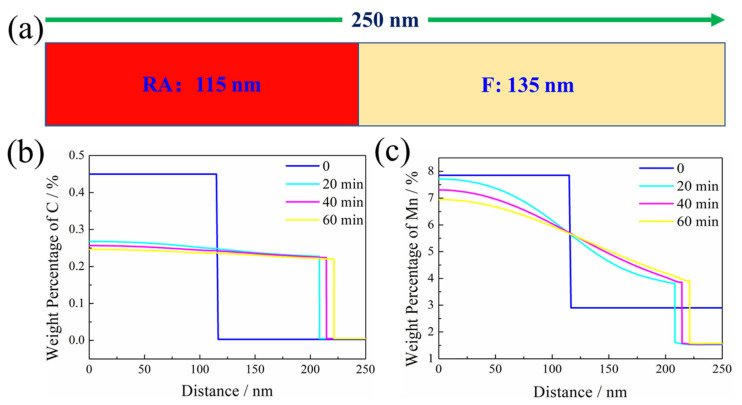
(**a**) Schematic diagram of the diffusion couple applied for DICTRA simulation of the SPA process. (**b**,**c**) Element concentration profiles of C and Mn near the γ/α interface, showing the moving γ/α interface.

After being subjected to the SPA process, the RA content decreased from 30% to 25.9% and 15.6% of SPA20 and SPA40, respectively. The decrease in RA content showed that part of it transformed into martensite, which was directly observed in Figure 7. The SPA process was simulated by DICTRA with MOB6 database (Figure 12a). The simulated result of the C and Mn concentration profile near the γ/α interface at 650 °C for 6 h is exhibited with a blue line in Figure 12b,c, which is regarded as the initial conditions for the SPA process. The γ/α two-phase interface migrated into ferrite during the SPA process, where the C and Mn content in ferrite had little variation between SPA20 and SPA40. The content of C and Mn decreased from the center to the edge, potentially indicating that martensite transformation appeared at the edge. This was consistent with the TEM observation that RA was surrounded by SM. The M_s_ temperature was estimated according to the following empirical formula [28]:

M_s_ (°C) = 539–423C–30.4Mn–7.5Si (2)

where C, Mn, and Si are their concentration in RA, wt.%. The schematic diagram of phase transformation is shown in Figure 1. Herein, the three-layer microstructures were obtained: ferrite (outer layer), SM (interlayer), and RA (inner layer).

In view of the optimal precipitation temperature range, all the (V, Mo)C particles precipitated during IT at 650 °C. Meanwhile, the mean particle size increased to 17.2 nm, showing the ripening appearing. In actuality, the ripening rate is high for single VC precipitation [29]; however, the ripening rate was relatively low because Mo was the effective element to inhibit coarsening in view of the larger atom size and lower diffusion velocity. The disclike (V, Mo)C was formed based on the Baker–Nutting orientation relationship in the matrix during IT [30], while few disclike particles were found at 750 °C for 20 and 40 min, implying that some of them dissolved during 750 °C preservation. For small particles, it was the edges of the discs that began to dissolve due to the specific interface energy varying with temperature [29].

### 4.2. Strengthening Contribution Analysis

Consisting of a mixed microstructure of ferrite, RA, and SM, the strengthening contribution calculation depends on the composite law, where both the content and single yield strength of ferrite, RA, and SM are considered. The yield strength of the studied steel obeyed the composite law of multiphase microstructure according to the following equation [6,31]:

(3) $$\sigma_{y}=V^{\gamma }\sigma_{y}^{\gamma }+V^{ferrite}\sigma_{y}^{ferrite}+V^{SM}\sigma_{y}^{SM}$$

where $V^{i}$ is the volume fraction of RA, ferrite, and SM; $\sigma_{y}^{j}$ is the yield strength of RA, ferrite, and SM.

- (V, Mo)C precipitation strengthening

The equilibrium content of (V, Mo)C particles is 0.00227 at 650 °C. There was no obvious coarsening of (V, Mo)C particles during the SPA process, so the mean size of 18 nm was used for the precipitation strengthening. (V, Mo)C precipitation strengthening occurred by the Ashby–Orowan dislocation looping mechanism, and it was calculated using the classical Ashby–Orowan equation, expressed as follows [32,33]:

(4) $$\sigma_{p}=\frac{0.8MGb}{2\pi \sqrt{1-\nu }l}\ln\left(\frac{d}{2.45b}\right)$$

where *M* and *v* are the Taylor factor and Poisson’s ratio, taken as 2.75 and 0.293. *G* is the shear modulus for low-carbon steel. *B* is the Burgers vector, taken as 0.2482 nm. Herein, it is assumed that (V, Mo)C particles were uniformly distributed in matrix. 𝑙 is the mean nearest-neighbor particle spacing [33]:

(5) $$l=\frac{1}{2}\sqrt{\frac{2}{3}}\left(\sqrt{\frac{\pi }{f}-2}\right)d$$

where *f* and *d* are the volume fraction and size of (V, Mo)C precipitation particles. The calculated precipitation strengthening is about 108 MPa. The (V, Mo)C precipitation strengthening in RA, ferrite, and SM are the same in hypothesis.

- Dislocation density and strengthening in ferrite and SM

**Figure 13:**
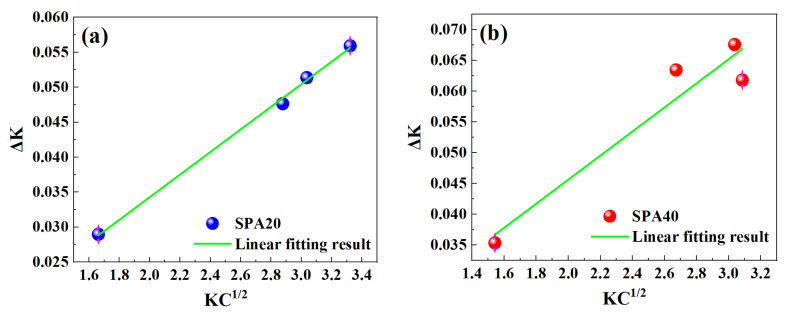
Peak broadening analysis: (**a**) SPA20 and (**b**) SPA40.

The dislocation density in ferrite is relatively low, such that it is hard to accurately estimate by the Williamson–Hall method using the XRD profiles. The dislocation density in ferrite is estimated to be about 1 × 10^14^/m^2^ [34]. The Williamson–Hall method usually overestimates the dislocation density, so Ungár et al. [35] developed a modified Williamson–Hall (MWH) method by accounting for the influence of the strain anisotropy. The dislocation density according to the MWH is as follows:

(6) $$\Delta K≅\frac{0.9}{D}+bM\sqrt{\frac{\pi }{2}\rho }\left(K{\bar{C}}^{1/2}\right)$$

where *D* is the crystallite size, *K* is the magnitude of the diffraction vector, *b* is the magnitude of the Burgers vector, and *ρ* is the dislocation density, $\bar{C}$ is the dislocation contrast factor, and *M* is a dimensionless constant. The dislocation density strongly depends on the slope of curves between Δ*K* and $K{\bar{C}}^{1/2}$ in Figure 13. Please refer to the literature for the detailed calculation steps [24,36,37]. The calculated dislocation densities of SPA20 and SPA40 are shown in Table 3.

The dislocation strengthening was estimated by the following equation [31]:

(7) $$\sigma_{\rho }^{i}=\alpha MGb\sqrt{\rho^{i}}$$

where *M* is the Taylor factor (4), *α* is the geometrical constant (0.25), *G* is the shear modulus of the material (80 GPa), *b* is the Burgers vector (0.2482 nm), and $\rho^{i}$ is the dislocation density in ferrite or SM. The calculated dislocation strengthening is displayed in Table 3.

- Yield strength of RA

The C content in RA is hard to accurately detect by TEM-EDS. The empirical formula of C concentration in RA is as follows [38]:

(8) $$a_{\gamma }=0.3556+0.00453x_{C}+0.000095x_{Mn}$$

where $a_{\gamma }$ is the lattice constant of RA, and $x_{C}$ and $x_{Mn}$ are C and Mn concentrations in RA. The calculated C content in RA is exhibited in Table 4, where Si, Mn, and Mo are tested by TEM-EDS.

The yield strength of RA is according to the following equation [6]:

(9) $$\sigma_{y}^{\gamma }=15.4\times \left(23x_{C}^{\gamma }+1.3x_{Si}^{\gamma }+0.94x_{Mo}^{\gamma }\right)+\frac{0.46}{\sqrt{d_{\gamma }}}+\sigma_{P}^{\gamma }$$

where $x_{C}^{\gamma }$, $x_{Si}^{\gamma }$, and $x_{Mo}^{\gamma }$ are the concentrations in RA. $d_{\gamma }$ is the RA size in nm (300 and 185 nm of SPA20 and SPA40, respectively). $\sigma_{P}^{\gamma }$ is the (V, Mo)C precipitation strengthening in RA. The yield strengths of RA in SPA20 and SPA40 were 362 and 355 MPa, respectively.

- Yield strength of ferrite

The yield strength of ferrite is determined by the following equation:

(10) $$\sigma_{y}^{ferrite}=\sigma_{0}+\sigma_{s}^{ferrite}+\sigma_{g}^{ferrite}+\sqrt{{\left(\sigma_{d}^{ferrite}\right)}^{2}+{\left(\sigma_{p}^{ferrite}\right)}^{2}}$$

where $\sigma_{0}$ is the lattice friction force, about 50 MPa; $\sigma_{s}^{ferrite}$, $\sigma_{g}^{ferrite}$, $\sigma_{d}^{ferrite}$, and $\sigma_{p}^{ferrite}$ are the solution, grain boundary, dislocation, and precipitation strengthening [39,40].

(11) $$\sigma_{s}^{ferrite}=4570X_{C}+60X_{\mathrm{Si}}+37X_{\mathrm{Mn}}+11X_{\mathrm{Mo}}$$

where *X_i_* is the element content of *i* in ferrite, wt.%.

(12) $$\sigma_{g}^{ferrite}=k_{y}D^{-1/2}$$

where *k*_y_ is the Hall–Petch parameter and *D* is the effective grain size [31].

The content of element *i* in ferrite and effective grain size (EGS) are displayed in Table 5, and the strengthening components of ferrite are shown in Table 6.

- Yield strength of SM

The strength of martensite is calculated in the same way as that of ferrite. The content of element *i* in SM and effective grain size (EGS) are shown in Table 7, and the strengthening components of SM are shown in Table 8.

- The calculated yield strength of the studied steel

According to Equation (3), the yield strength of the studied steel obeyed the composite law of multiphase microstructure. The calculated results are shown in Table 9.

The yield strength was 882 MPa and 1097 MPa of SPA20 and SPA40, respectively (Table 2). As the soaking time increased from 20 to 40 min, the yield and tensile strength increased by 215 and 180 MPa, while −20 °C impact energy, total elongation, and volume fraction of RA decreased by 3.4 J, 5.0% and 10.3%, respectively. The results show that RA had a more important effect on strength than toughness in such high-yield-strength steel, which did not agree with the report by Chen et al. [41]. It could be reasonably inferred that RA was so stable that the TRIP effect was hard to induce during impact tests, leading to the initial crack nucleation at the SM/ferrite boundary [6].

**Figure 14:**
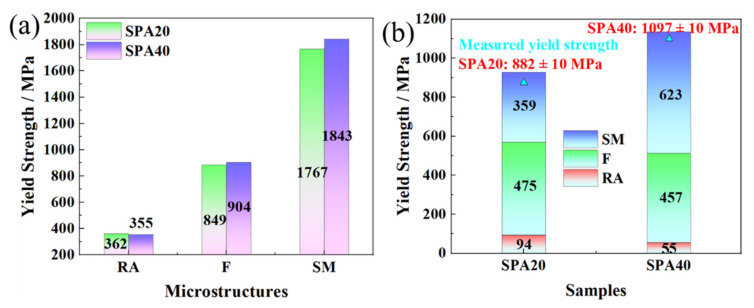
(**a**) Strengthening components of SPA20 and SPA40. (**b**) Strengthening contributions of SPA20 and SPA40 of the multiphase microstructures. F, RA, and SM represent ferrite, reversed austenite, and secondary martensite.

The strengthening increments of ferrite, RA, and SM are displayed in Figure 14. The calculated yield strengths of RA, ferrite, and SM were 355, 904, and 1843 MPa in SPA40, while they were 362, 849, and 1767 MPa in SPA20, respectively (Figure 14a). The high yield strength of ferrite was due to the ultrafine grain size transformed from martensite. Moreover, RA, ferrite, and SM provided the yield strengths of 94, 475, and 359 MPa of SPA20, and 55, 457, and 623 MPa of SPA40, respectively (Figure 14b). Although both RA content decrease and SM content increase increased the yield strength by about 215 MPa, they damaged total elongation by about 5.0%. However, the RA was stable such that the TRIP effect was hard to induce, leading to the small difference of −20 °C impact energy.

## 5. Conclusions

The short-time partial austenitization (SPA) process and V/Mo addition were introduced to optimize the phase transformation and final mechanical properties of 0.22C–5.24Mn steel. The main conclusions were as follows:

1. The initial microstructure was a dual-phase structure containing ferrite and RA after being intercritically tempered at 650 °C for 6 h, and the RA content was about 30%. Once subjected to the SPA process, the microstructure consisted of ferrite, RA, and SM, and the contents of them were 53.8%, 25.9%, and 20.3 for SPA20, and 50.6%, 15.6%, and 33.8% for SPA40.
2. The mean (V, Mo)C particle size was about 17.2 nm of IT650, while the mean particle size was about 18.0 nm of the SPA process. The morphology of the (V, Mo)C particles was disclike, while it tended to have near-spherical morphology after being subjected to the SPA process.
3. The yield strength increased by about 215 MPa from SPA20 to SPA40 of 1097 MPa, while there was acceptable shrinkage in total elongation from 19.0% to 14.0% and in −20 °C impact energy from 36.7 J to 33.3 J.
4. Protected by SM, the stable RA displayed a positive effect on yield strength, while it showed little effect on ductility and toughness. The ultrafine-grained ferrite and SM formed during the SPA process, jointly strengthened above 1000 MPa for SPA40. The (V, Mo)C provided a precipitation strengthening increment of about 108 MPa.

## Figures and Tables

**Table 1:** Volume fraction of RA, ferrite, and SM, respectively.

Samples	RA/%	Ferrite/%	SM/%
SPA20	25.9	53.8	20.3
SPA40	15.6	50.6	33.8

**Table 2:** Mechanical properties and RA contents of the steel subjected to the multistep heat treatment process.

Samples	Yield Strength /MPa	Tensile Strength /MPa	Total Elongation/%	Akv at −20 °C /J	V_γ_/%
SPA20	882 ± 10	1341 ± 11	19.0 ± 1.5	36.7 ± 2.3	25.9
SPA40	1097 ± 10	1521 ± 2	14.0 ± 1.0	33.3 ± 1.2	15.6

**Table 3:** Dislocation density and strengthening in ferrite and SM, respectively.

Samples	Dislocation Density (/m^2^) and Strengthening (MPa) in Ferrite	Dislocation Density (/m^2^) and Strengthening (MPa) in SM
SPA20	1 × 10^14^/149	1.375 × 10^15^/552
SPA40	1 × 10^14^/149	2.109 × 10^15^/683

**Table 4:** Nominal chemical composition in RA, wt.%.

Samples	C	Si	Mn	Mo
SPA20	0.6169	0.35	8.8	0.15
SPA40	0.5753	0.35	7.0	0.15

**Table 5:** Nominal chemical composition in ferrite and EGS.

Samples	C	Si	Mn	Mo	EGS/μm
SPA20	0.008	0.3	2.8	0.2	1.26
SPA40	0.008	0.3	2.5	0.2	1.19

**Table 6:** Different strengthening components of ferrite.

Samples	$\sigma_{0}$/MPa	$\sigma_{s}^{ferrite}$ /MPa	$\sigma_{g}^{ferrite}$ /MPa	$\sigma_{d}^{ferrite}$ /MPa	$\sigma_{p}^{ferrite}$ /MPa	$\sigma_{y}^{ferrite}$ /MPa
SPA20	50	160	490	149	108	884
SPA40	50	150	520	149	108	904

**Table 7:** Nominal chemical composition in SM and EGS.

Samples	C	Si	Mn	Mo	EGS/μm
SPA20	0.08	0.35	8.8	0.2	1.26
SPA40	0.08	0.35	7.0	0.2	1.19

**Table 8:** Different strengthening components of SM.

Samples	$\sigma_{0}$/MPa	$\sigma_{s}^{marteniste}$ /MPa	$\sigma_{g}^{marteniste}$ /MPa	$\sigma_{d}^{marteniste}$ /MPa	$\sigma_{p}^{marteniste}$ /MPa	$\sigma_{y}^{marteniste}$ /MPa
SPA20	50	714	490	553	108	1767
SPA40	50	648	504	683	108	1843

**Table 9:** The calculated and measured yield strength of SPA20 and SPA40, MPa.

Samples	$\sigma_{y}^{\gamma }$	$\sigma_{y}^{ferrtie}$	$\sigma_{y}^{SM}$	$\sigma_{y}$, Calculated Yield Strength	Yield Strength Measured Yield Strength
SPA20	362	884	1767	928	882
SPA40	355	904	1843	1135	1097

## Data Availability

Data are contained within the article.
